# Human Collaborative Localization and Mapping in Indoor Environments with Non-Continuous Stereo

**DOI:** 10.3390/s16030275

**Published:** 2016-02-24

**Authors:** Edmundo Guerra, Rodrigo Munguia, Yolanda Bolea, Antoni Grau

**Affiliations:** 1 Department of Automatic Control, Technical University of Catalonia (UPC), Barcelona 08034, Spain; edmundo.guerra@upc.edu (E.G.); yolanda.bolea@upc.edu (Y.B.); 2 Department of Computer Science, Universidad de Guadalajara (CUCEI), Guadalajara 44430, Mexico; rodrigo.munguia@upc.edu

**Keywords:** collaborative robotics, monocular SLAM, HRI, indoor mapping

## Abstract

A new approach to the monocular simultaneous localization and mapping (SLAM) problem is presented in this work. Data obtained from additional bearing-only sensors deployed as wearable devices is fully fused into an Extended Kalman Filter (EKF). The wearable device is introduced in the context of a collaborative task within a human-robot interaction (HRI) paradigm, including the SLAM problem. Thus, based on the delayed inverse-depth feature initialization (DI-D) SLAM, data from the camera deployed on the human, capturing his/her field of view, is used to enhance the depth estimation of the robotic monocular sensor which maps and locates the device. The occurrence of overlapping between the views of both cameras is predicted through geometrical modelling, activating a pseudo-stereo methodology which allows to instantly measure the depth by stochastic triangulation of matched points found through SIFT/SURF. Experimental validation is provided through results from experiments, where real data is captured as synchronized sequences of video and other data (relative pose of secondary camera) and processed off-line. The sequences capture indoor trajectories representing the main challenges for a monocular SLAM approach, namely, singular trajectories and close turns with high angular velocities with respect to linear velocities.

## 1. Introduction

The collaboration between humans and robots has become one of the fastest growing research fields in last years. While traditionally this collaboration was focused on industrial environments, where repetitive task would be performed by both robots and humans to produce goods and services, the increased accessibility—in terms of availability and cost—of the new mobile robotic systems has greatly expanded the possibilities of the human-robot interaction field (HRI) [1]. Collaborating in rescue operations, exploration tasks, disabled person assistance, or simply guidance in urban environments, with each passing year the HRI field leads to new application scenarios relying more on the autonomous capacities of robotic devices.

One of the main requirements to achieve real autonomous functionality in mobile robotic devices is the ability to understand the spatial relations with the environment, which can be divided into two different problems: (i) understanding the structure of the environment and the relations between the elements that compose it, known as mapping, and (ii) to be able to interpret the relations between the robotic device and those elements and environment, thus, locate the robot. These two dual problems are currently treated as a single problem, known as simultaneous localization and mapping (SLAM), or concurrent mapping and localization (CML). The most widely known approach to the SLAM problem is based on the Extended Kalman Filter (EKF), as it enabled the main breakthrough [2], where for the first time both aspects of the SLAM were formulated as a single convergent recursive estimation problem. Generally, any given SLAM [3,4] technique will rely on exploring the environment with one or more sensors, of proprioceptive or exteroceptive nature, as the robotic device moves through it, building a map with the data obtained from the sensors, and using this map to localize the robot in the environment. To fulfill these objectives exteroceptive sensors (camera, laser range finder, sonar…) are normally more useful, as the data they provide is better for describing the environment and surroundings, while the proprioceptive sensors (encoders, inertial sensors, magnetometers…) usually provide odometry and other data related exclusively to the robot.

One of the most widely used sensors in robotics research is the camera. The consumer push for digital cameras has led to the development of readily available, really cheap and reliable devices, which makes them much more accessible than any other kind of sensor. For example most of the robots specialized in social interactions within the HRI field deploy one or more cameras [5]. The camera itself, as a sensor, is able to produce great amounts of data, which within the SLAM context can be used to deal with several of the key issues after processing the image data with computer vision techniques. In [6,7] several other sensors are discussed within the SLAM framework, comparing their strengths and weaknesses against the use of monocular cameras, beyond the availability and accessibility of such sensors.

The utilization of cameras within SLAM procedures has its own challenges. The main issue is that standard cameras are essentially bearing-only sensors: while they can provide data about the luminosity and colour of a given point, spatially only the direction of the point is known, not the distance to it. As such, to compute the distance of a point to a camera sensor, two different images must be considered, and there must be separation, known as parallax, between the camera sensors: two cameras can capture the same point simultaneously, from different points of view, producing an instantaneous depth estimation; or a single moving camera, which captures the same point from different points of view in different time instants. The first case generally leads to the classical stereo approach [8,9], where epipolar geometry [10] is exploited within a calibrated camera rig where several restrictions must be satisfied allowing at the same to optimize data association [11]. On the other hand, using a single camera leads to monocular SLAM, which is a much more complex problem, especially the 6-degree of freedom (DoF) variants. Many of the monocular SLAM techniques are based on or derived from the EKF approach, and they present diverse ways to deal with the inability to achieve instant parallax to estimate depth. Some of breakthroughs in monocular SLAM were initially based on EKF, like the first monocular SLAM real-time approaches [12], the development of the inverse depth (I-D) feature parametrization model [13], and several loop-closing and large map management techniques [4].

Other approaches to monocular SLAM rely on techniques taken from the structure-from-motion (SfM) approach [14], the analogous problem in computer vision. Originally SfM problems were conceived to be solved off-line, and thus generally relied on non-linear global optimizations. These methods have been adapted and generated several approaches to monocular SLAM known as keyframe methods [15]. These techniques generally are based on bundle adjustment to produce accurate results [16], but they require a really big computational budget with heavy energy consumption [17]. Several approaches have solved these issues within the cloud robotics field, where a good strategy consists of separating the SLAM problem into two parallel threads, like PTAM [18]. Those algorithms can be optimized by moving computationally heavy processes to a remote server [19]. This kind of implementations can deal with really complex problems, like multiple robot SLAM and real-time map optimization thanks to the possibilities opened by the introduction of cloud computing. In [20] the architecture presented offered scalability capabilities, thus the cloud computing server could increase its computing capabilities. Anyway, enjoying these advantages require heavy infrastructure investments and continuous access to the network where the computing servers are deployed. As such, they can be inconvenient within a fully autonomous robotic context in unknown environments.

In a HRI collaborative context, the SLAM problem has been studied by several works in the domains of emergency response and companion/assistant robotics. While the specific properties in each domain may vary according to the application, the need for indoor and outdoor SLAM techniques where the human component is present as a key factor is clear. In [21], for example, large areas are explored by a wide group of persons and robots, where the human carry a wearable device, while in [22] a human explores and maps a building while carrying a mapping robotic device. Introducing HRI within the classical SLAM framework usually means increased complexity, like having to deal with dynamic objects in the vicinity and increasing the multimodal range of sensors. All these issues have responses in the SLAM research, thus it is better to concentrate on the new possibilities, such as trying to improve the depth estimation, or overcome other challenges. Thus, exploratory HRI opens the door to improve known mapping techniques exploiting the opportunities provided by the human component. With that end, an initial approach to monocular SLAM with collaborative perception based on HRI was presented in [23], where the delayed inverse depth monocular SLAM (DI-D SLAM) [6] with highest order hypothesis compatibility test (HOHCT) validation [7] was modified so that with the help of a secondary camera, the delayed feature initialization process could accept features with instantly estimated depth.

In this work, a new complete approach to collaborative SLAM is presented. Previous work [23], which introduced the pseudo-stereo approach only during feature initialization (but not the EKF estimation process), has been revised and expanded, so that the benefits of the secondary camera are used into the EKF estimation and correction. In Section 2, the basics of the technique presented in [23] are briefly described, and discussed, as a basis to introduce the proposed improvements. Section 3 deals with the contributions added to the approach, which now supposes a full collaborative SLAM approach, where the data obtained from the auxiliary sensor is exploited along the whole EKF SLAM algorithm, without being limited to the initialization process. Furthermore, the initialization process has been improved, with a new inverse observation model and its respective Jacobian, which models with more accuracy the errors, and so the covariance of new features. Section 4 briefly describes the experimental setups, while Section 5 details and discusses the results of the experiments and simulations done. Finally, an analysis of the results concludes this work, with discussion about the possible directions to follow in future research.

## 2. Monocular SLAM with Cooperative Feature Initialization

**Figure 1:**
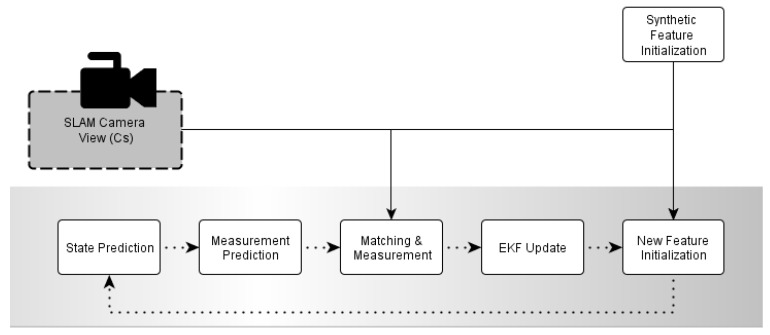
Delayed inverse-depth (DI-D) monocular EKF-SLAM.

The general monocular EKF-SLAM procedure is based on detecting points of interest, chosen between those to be considered landmarks and introduced into the EKF. Those landmarks will be tracked along the different video frames and their pose and camera odometry will be estimated. The estimation process is based on probabilistic filtering. An initial prediction step makes a prediction of the movement, and a further update (namely also a correction step) and compares the predicted observations obtained according to the movement prediction with actual observations from the sensor (Figure 1). While the undelayed approaches try to choose the points to become landmarks and initialize them when they have been seen for the first time, the delayed approaches generally rely on obtaining a previous depth estimation. These two types of strategies define many characteristics of the SLAM procedures. For instance, undelayed approaches try to use point features as landmarks just after they have been seen, then these points are quickly introduced into the filter accepting many outliers that have to be validated in a later refinement [24]. This validation step generally removes many points, thus an undelayed approach needs to add constantly new feature points. On the other side, delayed approaches track and estimate the points before using them. Although a validation process is still required, the used landmarks are generally more stable and reliable. This way, while the undelayed approaches put less effort per landmark during initialization, the performance is similar compared with delayed approaches [7], using less points and with less computationally burdensome validation algorithms.

In [23], a new feature initialization methodology was proposed in the context of the DI-D monocular SLAM [6], exploiting the chances offered by a second bearing-only camera sensor. This camera is worn by a human accompanying the robot during the SLAM procedure and it is considered that this new camera (C_f_) have a known pose (T_c_) w.r.t. the main monocular SLAM camera (C_s_). The sensory system of the robot locates the distance and orientation of such a human respect the robot [5]. Mathematically, this approach kept the EKF architecture of its predecessors [6,23]. The augmented state vector, $\hat{x}$ (Equation (1)), denotes the pose of the camera C_s_ (${\hat{x}}_{v}$) and the feature map (**Ω**). The first part of this column vector $\hat{x}$ contains a vector ${\hat{x}}_{v}$ that represents the robotic camera device, describing its pose (**r***^WC^*, **q***^WC^*) and speeds (**ν***^W^*, **ω***^W^*) (Equation (3)). The map estimation **Ω** is represented by a set of features (Equation (2)) where each feature ${\hat{y}}_{i}$ is stored as a vector which models the estimated landmark position (Equation (4)) according to the inverse-depth model [13]. Coordinates *xi, yi, zi* are the optical centre of the camera; *θi, φi*, are azimuth and elevation for the ray which traces the feature point; depth *r_i_* to the feature is coded by its inverse*: ρ_i_ = 1/r_i_* , see [13]:

(1) $$\hat{x}={\left[{\hat{x}}_{v},\Omega \right]}^{T}$$

(2) $$W={\left[{\hat{y}}_{1},...{\hat{y}}_{n}\right]}^{T}$$

(3) $${\hat{x}}_{v}={\left[\begin{array}{cccc} r^{WC} & q^{WC} & v^{W} & \omega^{W} \end{array}\right]}^{T}$$

(4) $${\hat{y}}_{i}={\left[\begin{array}{cccccc} x_{i} & y_{i} & z_{i} & \theta_{i} & \varphi_{i} & \rho_{i} \end{array}\right]}^{T}$$

As a metric scale initialization is required to estimate parallax in the DI-D monocular SLAM approach, a procedure to introduce a set of initially known features artificially was present, estimating camera C_s_ pose by the PnP problem [25]. This characteristic was kept in the pseudo-stereo feature initialization approach, to allow operation without initial C_s_ and C_f_ views overlap, enabling direct comparison of the results with and without pseudo-stereo operation. After that, the general process of monocular EKF-SLAM was followed by a prediction phase using a constant-acceleration motion model for the camera, and static mapping assumption for the landmarks. An active search strategy was used for measurement and matching [26], employing the zero-normalized cross-correlation to find the better match for each landmark in a new frame. Once the prediction were matched, a batch validation test of the matching pairs found was used, the HOHCT [7], and the state and covariance corrected with the EKF update step.

The main contribution of this pseudo-stereo feature initialization approach relied on the presence of a secondary monocular sensor (C_f_) worn by a human which satisfied three conditions:

- It produces data of similar nature to C_s_ (like being a similar camera).
- Its pose with respect to C_s_ is known at any time (with an estimated error).
- During some frames C_f_ would observe the same scenes as C_s_.

These three conditions, frequently satisfied in collaborative robotics environments, allowed speeding up the feature initialization process, reducing the number of required observations along video frames of an interest point. Instead of the DI-D initialization [6], under the collaborative pseudo-stereo initialization a feature would be initialized without delays with its stereo depth estimation. Like in the undelayed approaches this initialization is done with the actual depth estimation, not a heuristic value. As features could be initialized instantly, the risk of spurious/dynamic points increased, thus the HOHCT [7] was chosen as the data validation methodology. This methodology allows maintaining the EKF architecture providing a good performance, as the landmarks initialized through stereo also keep a good depth estimation, which gives HOHCT an edge over other methods.

**Figure 2:**
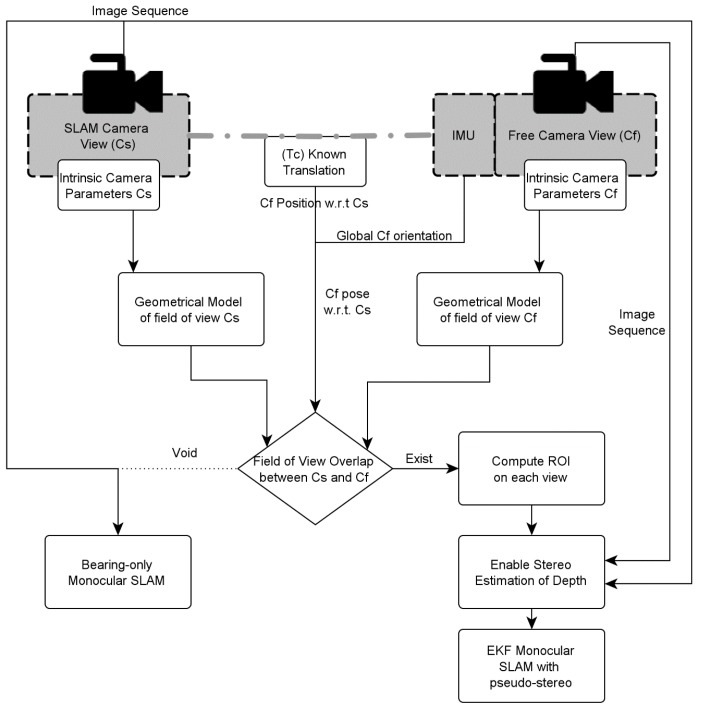
Detection of uncalibrated non-continuous stereo, with geometrical modelling and estimation of the coincidental area.

To avoid searching matches between images from C_f_ and C_s_ when the cameras were not looking at the same region of space, a procedure based on geometrical modelling was used to predict whether there would be overlap between the cameras and estimate a region of interest (ROI) [23]. The procedure (Figure 2) was based on modelling the fields of view of both cameras as pyramids, using the calibration data and an arbitrary value for depth, derived from a heuristic value for the minimal desired parallax angle. The pose between cameras is assumed to be known within a given error, through combination of an inertial measurement unit (IMU) and other sensors/devices (like a laser range finder, external measurement to obtain ground truth).

Thus, if the procedure detected that both cameras were looking at the same area within a limited depth, it was considered that they were observing the same parts of the environment. Under this hypothesis, the initialization process was speeded up by searching matches in C_f_ image for the candidate landmark points tracked in C_s_ image using SURF [27] descriptors. The candidates which presented a match in C_f_ were preferred to be initialized, as they could provide depth information instantly, simplifying the feature initialization.

## 3. Full Collaborative Monocular SLAM with Feature Measurement

**Figure 3:**
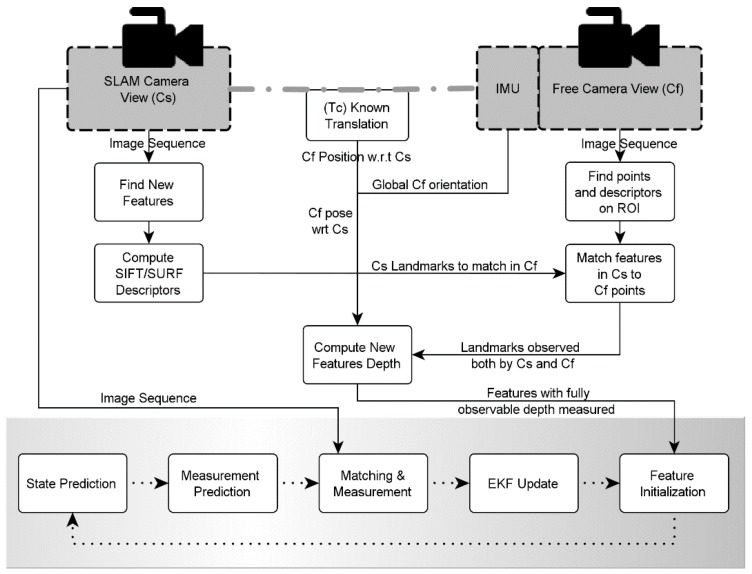
Monocular EKF-SLAM with stereo-enhanced feature initialization.

The non-constant stereo approach for landmark initialization (as seen in Figure 3) has been refined and extended, introducing a new Jacobian computation procedure to update the covariance matrix when introducing new data into the augmented state vector. But the most relevant improvement is the introduction of the non-constant stereo depth estimation during the measurement step.

### 3.1. Collaborative SLAM in Measurement

In [23] the inverse depth parametrization is still the basis for the observation model **h***^c^* (Equation (5)). As the inverse depth model **h***^c^* produces 3D world coordinates with respect to the camera, *h_x_, h_y_, h_z_* coordinates are projected into the camera and the obtained undistorted pixels *(u_u_, v_u_)* (Equation (6)) are distorted to produce feature predictions within camera pixel-coordinates space:

(5) $$h^{c}=\left[\begin{array}{c} h_{x}\\ h_{y}\\ h_{z} \end{array}\right]=R^{CW}\left(\left[\begin{array}{c} x_{i}\\ y_{i}\\ z_{i} \end{array}\right]+\frac{1}{\rho_{i}}m\left(\theta_{i},\varphi_{i}\right)-r^{WC}\right)$$

(6) $$h=\left[\begin{array}{c} u_{u}\\ v_{u} \end{array}\right]=\left[\begin{array}{c} u_{0}\frac{f}{d_{x}}u\\ v_{0}\frac{f}{\rho_{y}}v \end{array}\right],\text{ where }\left[\begin{array}{c} u\\ v \end{array}\right]=\left[\begin{array}{c} \frac{h_{x}}{h_{z}}\\ \frac{h_{y}}{h_{z}} \end{array}\right]$$

These predictions in pixel coordinates are used during the measurement and correction processes in the update step of the EKF methodology as well as the predicted observations for the landmarks. This matching result is used as measurements obtained from the sensors (using the active search method). Instead, the proposed approach works with predictions and observations both in pixel space and in real world coordinates, that is, the stereo data sparsely available is used not only for feature initialization, but also for estimation and measurement. As such, the observation measurement and data association process, which were earlier done in one step thanks to the active search technique, now implies several more steps to support the measurement of the landmarks in the world space (w.r.t. the C_s_ camera) when available.

**Figure 4:**
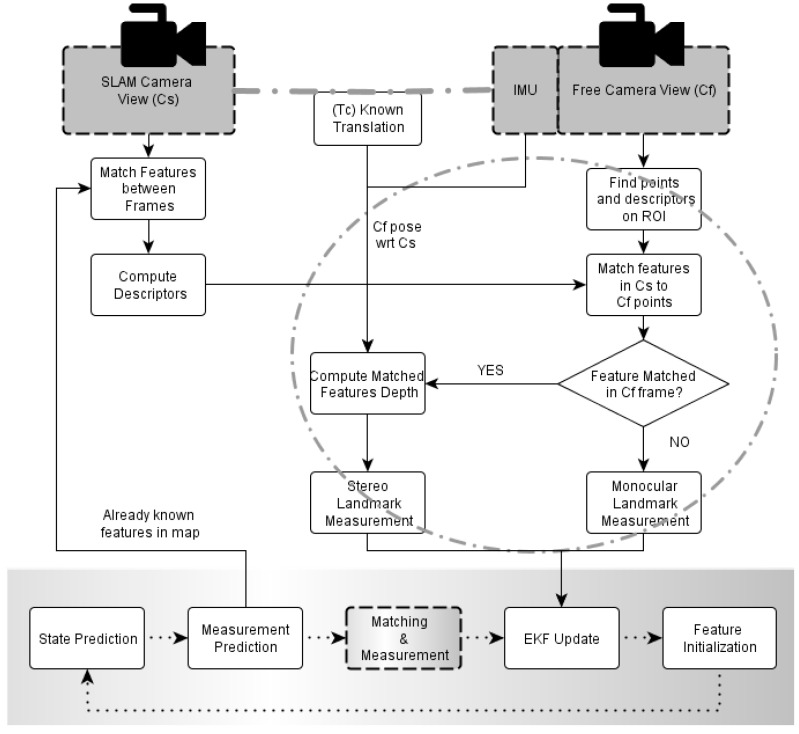
Monocular EKF-SLAM with complete pseudo-stereo, including measurement and matching.

The process with the new steps is shown in Figure 4. The known features are matched through active search between frames in the sequence obtained with camera C_s_, to keep tracking accuracy consistent only when monocular data is available. For those features lying in the region determined by both cameras overlap, C_s_ and C_f_, an additional descriptor, such as SURF, is computed. In the image obtained from C_f_, points of interest and their descriptors are searched within the determined region of interest. These points are then matched with the SURF descriptors from the known features. Those known features without a match in C_f_ will be treated as only-bearing features, using the pixel position on image as measurement, and therefore the same well-known formulation will be used during the filter update step. Those features with a matching point in C_f_ are measured in terms of world coordinates, and thus a different approach will be required during the update step.

During the update step of EKF there are several matrices that must be built with a size fitting to the innovation vector **g**. This vector is essentially a stack with the residuals of the obtained observations and the predicted observations from known features (Equation (B5) in the Appendix B). As some features will be treated as observable through only-bearing data, while others will be considered as within fully observable space, **g** will change accordingly to: (i) the number of observed features, as always, and (ii) how these features are observed (as pixel coordinates or as Euclidean points in space).

The augmented state vector and the covariance matrix will remain the same, as the features are noted according to the inverse depth model. But the Jacobian matrices of the observation model, used on several equations (Equations (B3) and (B4) in the Appendix B), will change not only in size but in the way they are build. In previous works [6,7], as **h**^*c*^ (Equation (7)) is to be projected into C_s_ and distorted once in pixel coordinates, the unique relevant information was the bearing with respect to C_s_. This fact allows replacing Equation (5) with Equation (7) in order to simplify the symbolical computation of the Jacobian ∇*H*:

(7) $$h^{c}=\left[\begin{array}{c} h_{x}\\ h_{y}\\ h_{z} \end{array}\right]=R^{CW}\left(\rho_{i}\left(\left[\begin{array}{c} x_{i}\\ y_{i}\\ z_{i} \end{array}\right]-r^{WC}\right)+m\left(\theta_{i},\phi_{i}\right)\right)$$

This change allows for a simpler derivation of the required Jacobian for the bearing-only observation case, but as the coordinates are shifted, this step is not enough when the landmark is considered fully observable (as being seen by both cameras), and estimated in real space. Thus, to complete the new approach, a new Jacobian is computed considering the observation model to be composed only by Equation (5) without changes, and ignoring all the terms related to the projection and distortion, described in Equation (6).

### 3.2. Introducing New Landmarks into the Covariance Matrix

When a new landmark is introduced as a feature in the EKF state vector the data describing the landmark uncertainty and relations with previous estimations must be introduced into the covariance matrix. The general EKF SLAM methodology introduces the new data using Equation (8) as:

(8) $$P_{k}=\nabla Y\left(\begin{array}{cc} P_{k} & 0\\ 0 & R_{j} \end{array}\right)\nabla Y^{T}$$

Let *R_j_* be a diagonal matrix containing the error variance parameters of the sensor and the parameters stored for the new landmark, and let ∇*Y* be the Jacobian of the inverse observation model. The inverse observation model is used to compute the characterization of an observed landmark as an inverse-depth feature, using data from the sensors and the current estimates of the system. In an authors’ previous work [23], the features initialized through delayed initialization used the DI-D initialization process (seen at [6]), while those added through the stereo estimation used a classic monocular inverse-depth model, as proposed by Civera [24]. Though the impact in the algorithm is minimal, this fact supposed an underrepresentation of the uncertainty related to the secondary camera C_f_. In this present work, the matrix *R_j_* and ∇*Y* have been used to add features to matrix *P* (augmented state covariance matrix) accounting for the uncertainties derived from both cameras, having a total of 11 parameters to represent uncertainty instead of 2. This is achieved by replacing *R_j_ =* diag(σ_u1_^2^, σ_v1_^2^) with *R_j_’ =* diag(σ_u2_^2^, σ_v2_^2^, σ_u1_^2^, σ_v1_^2^, σ_xλ_, σ_yλ_, σ_zλ_, σ_q0λ,_ σ_q1λ_, σ_q2λ_, σ_q3λ_), where (σ_ui_^2^, σ_vi_^2^) denotes the pixel uncertainty for both cameras, (σ_xλ_, σ_yλ_, σ_zλ_) denotes the uncertainty of the position of camera C_f_, and (σ_q0λ,_ σ_q1λ_, σ_q2λ_, σ_q3λ_) denotes the uncertainty in camera C_f_ orientation quaternion. To accommodate all these new variables, a new Jacobian ∇*Y*‘ is introduced, which is formulated analogously to that described in [6] for delayed features.

### 3.3. Observability Enhancement Analysis

In this section an observability analysis is carried out. This analysis will show that the observability of the system is improved when non-continuous stereo measurements are incorporated into the system.

A system is defined as observable if the initial state *x*_0_ at any initial time *t_0_* can be determined given: (i) the state transition and observation models of the system, and (ii) observations *y*[*t_0_,t*] from time *t_0_* to a finite time *t*. When a system is fully observable, the lower bound of the error in the state estimation depends only on the noise parameters of the system and it is not reliant on initial information about the state. This concept has remarkable consequences in the context of SLAM.

**Figure 5:**
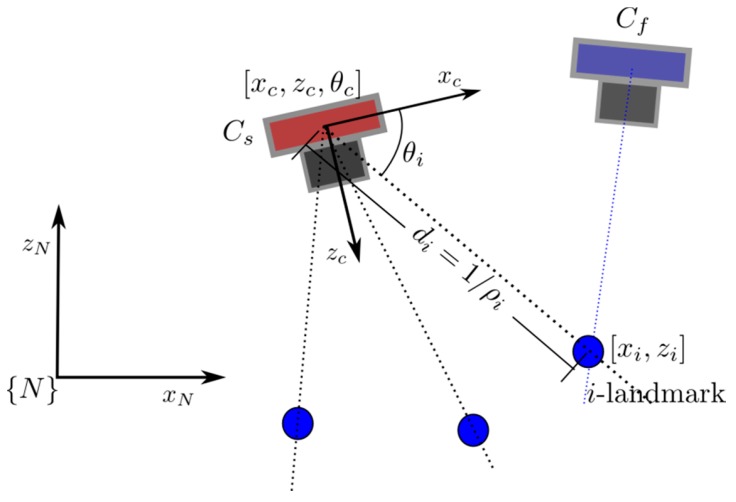
A 2-DoF simplified version of the proposed system used for performing the observability test.

In order to carry out the analysis, a simplified version of the proposed system is assumed (see Figure 5). Let consider the following unconstrained camera model ${\dot{x}}_{c}=f(x,u)$ for the camera C_s_:

(9) $$\begin{array}{l} {\dot{x}}_{c}=v_{x}\;{\dot{z}}_{c}=v_{z}\;{\dot{\theta }}_{c}=\omega_{z}\\ {\dot{v}}_{x}=V_{x}\;{\dot{v}}_{z}=V_{z}\;{\dot{\omega }}_{c}=\Omega \end{array}$$

Let *x_c_* = [*x_c,_ z_c,_ θ_c,_ v_x,_ v_z,_ ω_c_*] be the system state of camera C_s_. Let [*x_c,_ z_c,_* *θ_c,_*] represent the position and orientation of the camera, and [*v_x,_ v_z,_ ω_c_*] their first derivatives. In this model, it is assumed an unknown input *u* = [V_x_, V_z_, Ω] with linear and angular accelerations with zero-mean and known-covariance Gaussian processes. Also it is assumed that the camera C_s_ it is capable of detecting and tracking feature points coded with its inverse depth. In this case, the measurement process is modelled as:

(10) $$y_{i}=h_{\theta i}(x)=\arctan2\left(\frac{z_{c}-z_{i}}{x_{c}-x_{i}}\right)-\theta_{c}$$

where [*x_i_ , z_i_*] is the Euclidean position of the *i*-th feature coded by its inverse form. In this case:

(11) $$\begin{array}{l} x_{i}=\left(\frac{1}{\rho_{i}}\right)\cos(\theta_{i})+x_{0i}\\ z_{i}=\left(\frac{1}{\rho_{i}}\right)\sin(\theta_{i})+z_{0i} \end{array}$$

The state of the *i*-th feature *w_i_* is defined by *w_i_* = [*x_0i_, z_0i_, θ_i_, ρ_i_*] where [*x_0i_, z_0i_*] is the position of the camera C_s_ when the feature was detected for the first time, *θ_i_* is the first bearing measurement, and *ρ_i_* = 1/*d* is the inverse of the feature depth *d*. Since [*x_0i_, z_0i_, θ_i_*] is directly given when the feature is initialized, it is assumed that the system state $\hat{x}$ to be estimated is made up by the state of the camera C_s_ and the inverse depth of the features, as $\hat{x}=[{\hat{x}}_{c},\;{\hat{\rho }}_{1},\;{\hat{\rho }}_{2},\;...,\;{\hat{\rho }}_{n}]$.

Pseudo-stereo measurements provide information about the feature depths, and those measurements will be available only when some overlapping exists in the field of view (FoV) of both cameras C_s_ and C_f_. Thus, a pseudo-stereo measurement of the *i*-th feature is modelled as:

(12) $$y_{i}=h_{\rho i}(x)=\frac{1}{\rho_{i}}$$

Hence, for *n* landmarks being measured by the camera C_s_, and from which *m* pseudo-stereo measurements (*m*≤ *n*) are available, the system output is defined as *y* = [*h_θ1_, …, h_θn_, h_ρ1_,…, h_ρm_*]^T^.

In [28], a nonlinear system is demonstrated to be *locally weakly observable* if the observability rank condition (rank (O) = dim(*x*)) is verified. The observability matrix O is computed from:

(13) $$O={\left[\frac{L_{f}^{0}{\left(h_{\theta 1}\right)}^{T}}{\partial x}\;\frac{L_{f}^{1}{\left(h_{\theta 1}\right)}^{T}}{\partial x}\;...\;\frac{L_{f}^{0}{\left(h_{\theta n}\right)}^{T}}{\partial x}\;\frac{L_{f}^{1}{\left(h_{\theta n}\right)}^{T}}{\partial x}\;,\;\frac{L_{f}^{0}{\left(h_{\rho 1}\right)}^{T}}{\partial x}\;...\;\frac{L_{f}^{0}{\left(h_{\rho n}\right)}^{T}}{\partial x}\right]}^{\;T}$$

Let *L^i^_f_* (*h*) be the *i*-th order Lie Derivative [29] of the scalar field of the measurement *h* with respect to the vector field *f*. Note that in Equation (13), the zero-order and first-order Lie derivatives have been used for each bearing measurement *y_i_* = *h_θi_*(*x*). For pseudo-stereo measurements *y_i_* = *h_ρi_*(*x*) only the zero-order Lie derivative has been used.

Specifically and for the sake of demonstration, authors have investigated the case when bearing measurements *y_i_* = *h_θi_*(*x*) of four landmarks are available, that is $\hat{x}\;=\;[{\hat{x}}_{c},{\hat{\rho }}_{1},{\hat{\rho }}_{2},{\hat{\rho }}_{3},{\hat{\rho }}_{4}]$ and dim($\hat{x}$) = 10. The observability matrix O was computed using the MATLAB symbolic toolbox for three cases: (i) no pseudo-stereo measurements are available, (ii) one pseudo-stereo measurement is available, (iii) two pseudo-stereo measurements are available:

- *First case*, when there is no availability of pseudo-stereo measurements the rank(O) = 8, and therefore there exist two non-observable modes in the system.
- *Second case*, with a unique pseudo-stereo measurement, rank(O) = 9, and hence, one more mode becomes observable.
- *Third case*, when two pseudo-stereo measurements are available, rank(O) = 10, and therefore the system becomes fully observable.

The above result is interesting because it demonstrates that the system will become fully observable even if only a subset of the landmarks seen by camera C_s_ is also detected by camera C_f_. Furthermore, as it could be expected, the observability of the system is improved by incorporating pseudo-stereo measurements.

## 4. Experimental Setup

The technique proposed in this paper has been tested with simulations and real experimental data, which have been implemented and executed in MATLAB^®^. The set of data sequences used to test this approach has been captured using several sensors, with recording and synchronization through ROS. ROS middleware provides the software package *gmapping* used to compute the ground truth trajectories with the robot sensors. The video sequences capture took place in semi-structured indoor environments with a pair of Logitech C170 webcams, which each produced 10 frames per second. These sequences were processed in order to reduce the resolution to manageable terms (720 × 480 pixels), and convert from colour to grayscale, reducing the computational effort and enabling the utilization of standard feature detectors and descriptors.

**Figure 6:**
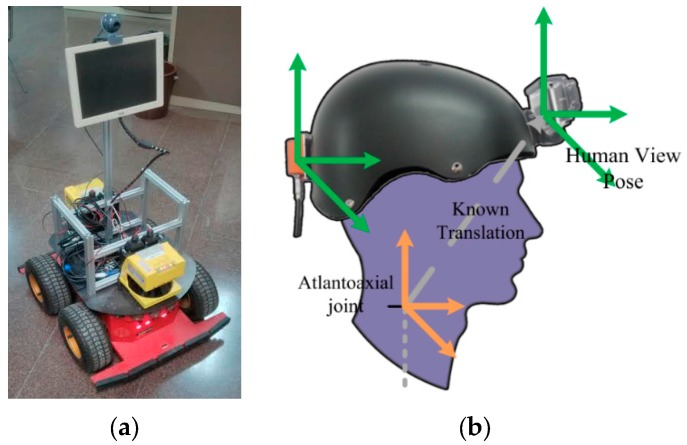
The (**a**) robotic platform and (**b**) wearable device used to capture data.

Each experimental sequence captures a collaborative exploration of an environment, where the C_s_ camera is assumed to perform the mapping and localization tasks from the point of view of a robotic platform (see Figure 6). This robotic device is supposed to accompany a human who wears another camera, C_f_. The robotic device tasks were performed by a robot based on the Pioneer 3 AT, which deploys a pair of laser range finders Leuzer RS4-4 in addition to the camera [30]. This allows producing an estimated ground truth and, using an IMU deployed with the camera C_f_, it is possible to estimate the pose between C_f_ and C_s_. To that end the position of the human is detected with the range finder lasers, and making several assumptions (as knowing the person’s height and that will remain standing), the final position of the camera C_f_ is estimated and fused with the pose from the Attitude and Heading Reference System (AHRS).

This pose of the camera worn by the human respect to the SLAM camera is not assumed to be perfectly known. Instead, it is considered that a "noisy" observation of the pose of C_f_ respect C_s_ is available by means of the methodology described above. The inherent error to the observation process is modeled assuming that the observation is corrupted by Gaussian noise. An alternate method could be used for computing the relative pose of C_f_, for instance using different sensors. However, even with the use of a more reliable methodology the errors would not be completely eliminated. For reference, the main specifications of the sensors used are found in Appendix C.

## 5. Results and Discussion

The whole data fusion process proposed, together with the inclusion of additional data during both the EKF update step and the initialization of new landmarks, has been evaluated and tested within indoor environments. The tests have shown clearly how monocular SLAM approach can greatly benefit from the sparsely distributed in time data provided by the freely moving camera, C_f_. This C_f_ camera, acting as an auxiliary monocular bearing-only sensors deployed as a wearable device by a human, helps composing a “virtual sensor” with instant depth estimation capabilities, creating a new monocular SLAM approach with greater accuracy and reliability.

In the context of indoor mapping, the SLAM problem presents several specific challenges related to the usual morphology of buildings aimed at human usage. This kind of buildings usually present corridors and other structures where the dominant movement to cross them is forward advance along the depth axis of the camera, or pretty tight turns. By contrast, monocular SLAM approaches rely most of the time on sideways movement to avoid the singular-forward advance-trajectories, and avoid close turns, expanding them to long curves. Another recurrent issue, not only in indoor visual mapping, but in structured environments, is the appearance of texture, repeated patterns, or simply, similarly looking objects, which raise the challenge of the data association problem from “looking for a good match” to “discriminating the correct match between the good ones”.

### 5.1. Experiments with Simulation

**Figure 7:**
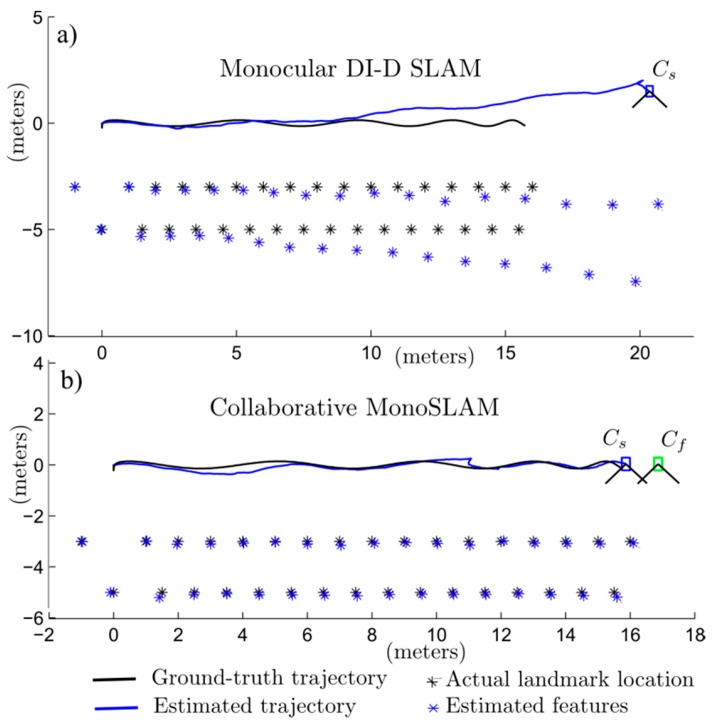
Estimated map and trajectory obtained with (**a**) monocular DI-D SLAM and (**b**) collaborative monocular SLAM.

To validate the gain in terms of depth estimation, the 2-DoF simplified version of the proposed system which is described in Section 3.3, has been simulated, assuming that it will be moving in a trajectory approximately parallel to a wall with known points that can be used by the system as visual landmarks (see Figure 7). The orientation of the camera varies a few degrees, but it is maintained approximately perpendicular to the landmarks. In simulations it is assumed that camera is able to track without error all the landmarks inside its field of view. The objective of the experiment is to evaluate the benefits obtained from incorporating pseudo-stereo measurements into the system for short periods of time.

The following parameters were used in simulations for the SLAM camera C_s_: noise for angular measurements σ_Cs_ = 1°, field of view FoV = 70°. Pseudo-stereo measurements, which are available when there is some overlap of the FoV of both cameras C_s_ and C_f_, are emulated by assuming highly-noisy measurements of range and bearing. In this case, the noise for angular measurements is σ_Csf_ = 6°, and the noise for range measurements is σ*_r_* = 0.5 m. In the simulated experiments, the camera was moved approximately 14 m during 30 s of simulation time. For two periods of time, from the second 8 to the 9, and from the second 17 to the 19, it was assumed that pseudo-stereo measurements were available for being incorporated into the system.

Figure 7a shows the results obtained from a run of the simulation when no pseudo-stereo measurements are available (pure monocular DI-D SLAM). For the pure monocular SLAM only a camera is needed (C_s_). In the experiment the camera was taken further than the ground truth but no recovery was done. In this case it can be clearly appreciated a huge drift in the error of the estimated map and trajectory. In this plot, also note the degradation of the metric scale in estimations. Figure 7b shows the results obtained when pseudo-stereo measurements are incorporated into the system. For this experiment both cameras were needed, the SLAM camera C_s_ and the free camera C_f_. It is worth noting that only two short periods, when pseudo-stereo measurements were available, were enough for improving considerably the estimation. In both experiments the length of the ground truth is the same although the length of *x*-axis is different, this is to fit the extra length of pure monocular SLAM camera trajectory.

**Figure 8:**
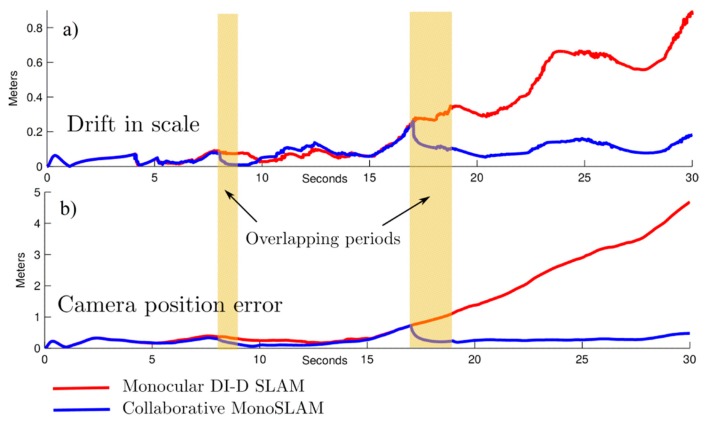
Average MAE (mean absolute error) computed (**a**) for drift in scale, and (**b**) for camera position. For the results obtained with collaborative SLAM, the translucent rectangles indicate periods of time during of which pseudo-stereo measurements are available. Note how MAE is minimized just after that the above occur.

Figure 8 shows the average MAE (mean absolute error) for drift in scale (Figure 8a) and camera position (Figure 8b), obtained after 20 Monte Carlo runs of simulation. For evaluating the degradation of the metric scale, the following measurement function was used:

(14) $$s=\left\|1-mean\left(\frac{d_{i}}{{\hat{d}}_{i}}\right)\right\|$$

where *d_i_* is the actual depth of a feature, and the set *i* = {1,2,..*n*} represents the features seen by the camera at that moment. The variable ${\hat{d}}_{i}$ is the estimated depth for the same *i*-th feature. In this case a relation $d_{i}$/${\hat{d}}_{i}$ = 1 represents that the metric scale of a feature has been perfectly recovered. The above expression is only computed for those features with a small covariance where it is assumed that the estimated depth has converged. Hence, in Equation (14), small values of *s* imply that the metric scale is better held in the system.

In Figure 8 it can be clearly appreciated how both the drift in the metric scale and the error in position, are minimized just after the inclusion of pseudo-stereo measurements into the system. Note that the above effect is especially notorious during the second period of cameras overlapping.

**Figure 9:**
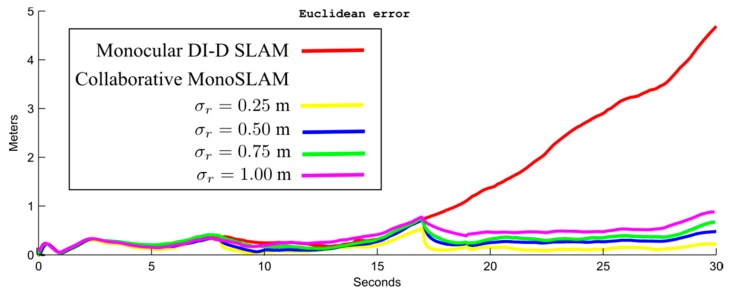
Average MAE (mean absolute error) computed from camera position for different values of uncertainty σ_r_ in pseudo-stereo measurements. Note that even with a considerable value of uncertainty in estimates of depth provided by the pseudo-stereo rig, the MAE is well bounded compared with the purely monocular approach.

Figure 9 shows the average MAE in camera position when parameter σ_r_ varies. The objective is to investigate the effectiveness of the proposed approach for different values of uncertainty in pseudo-stereo measurements. As it can be appreciated from this experiment, even, if very noisy pseudo-stereo measurements are incorporated into the system, the error in estimates can be considerably mitigated.

**Figure 10:**
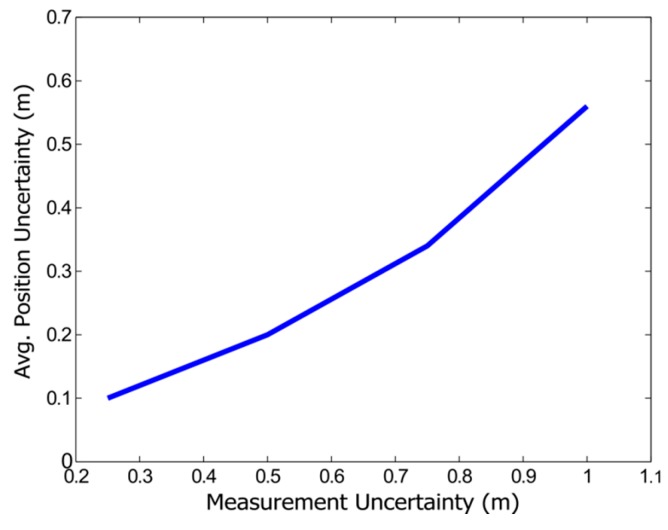
Relationship between depth measurement uncertainty and average MAE position uncertainty.

In order to study the relationship between the measurement uncertainty and the camera trajectory estimation uncertainty, the average MAE for the trajectories with varying σ_r_ was computed. Figure 10 shows different average MAE for the trajectories, whose measurement uncertainty varies between 0.25 and 1 m. The plot shows a strong correlation between the uncertainties between the measurement process and the estimation of the trajectory. Thus, it can be concluded that an improvement in the accuracy of the depth estimation should provide a strong improvement in the general estimation of the map, reducing the uncertainty inside the EKF.

### 5.2. Singular Trajectories and Movements

A set of frontal advancing sequences were captured through ROS and processed offline with the proposed technique. During the recording, the exploring team composed of a human and the robotic platform travels through a straight corridor. Note that under movements aligned with the camera depth axis, only really long trajectories produce enough parallax to enable landmark depth measurement, thus these are the worst cases for delayed monocular approaches, on which this work is based. At the same time, long movements generally produce that the relative perceived size of the elements on the environment tend to vary, thus inducing scale variability, which combined with reflective phenomena and possible repetitive textures, both robustness and reliability are reduced. Some works, like [6], exploit distant features, initializing them with heuristic values, and rely on them to achieve and reduce the effects of noise on orientation estimation and improve stability. Though similar to this case, note that in singular movements, especially in corridors, most of the solid landmark candidates will be found as unreliable to be fully initialized under a delayed approach in a reasonable number of frames.

**Figure 11:**
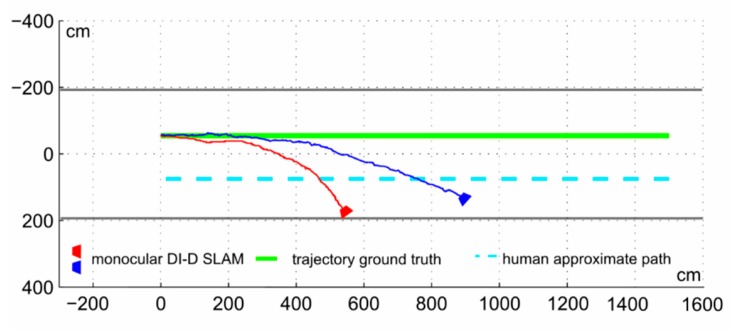
Worst (red) and average (blue) cases for standard monocular DI-D SLAM within a corridor in singular trajectory.

**Figure 12:**
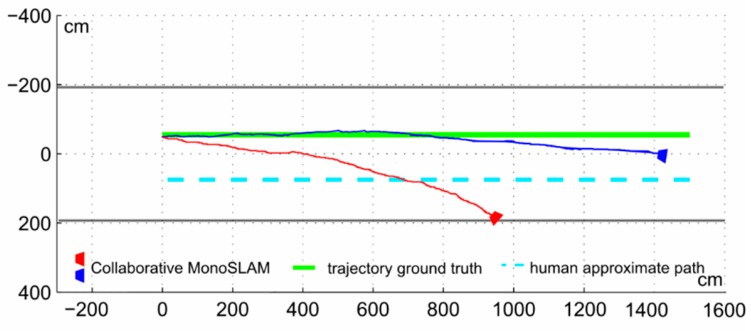
Worst (red) and average (blue) cases (same sequences as Figure 11) for collaborative monocular SLAM within a corridor in singular trajectory.

The battery of tests consisted in a series of ten sequences captured in similarly looking corridors, trying to obtain a 15-m trajectory map without using any of the classic large map management techniques [4]. The robot speed was adjusted to approximately match a walking person, about 1.5 m/s. Figure 11 and Figure 12 show the estimated odometry results (the sequence of camera optical center **r***^WC^* values) for two cases: worst scenarios (red) and average case scenario, with Figure 11 showing the trajectories for the monocular SLAM and Figure 12 those for the proposed collaborative SLAM.

In the worst case trajectory (red lines) both approaches underestimate the displacement and achieve a huge orientation error. Still, in the proposed approach errors are lesser, with almost double the distance advance along the depth camera axis, traveling almost 60% of the 15 m. For the blue trajectory (average case in both figures), the standard procedure manages to advance a notable 8.9 m. Nevertheless, in larger scenarios, the process is useless incurring in a noticeable orientation error supposing to be a straight trajectory. On the other hand, the proposed approach falls short of the target by less than 1m with minimal orientation error (about 9.5°). Average error metrics from the whole 10 sequences set are found in Table 1.

The accumulated and instantaneous position errors are computed according Equations (15) and (16) respectively, with the averages for all the 15 m long experiments shown on Table 1. Let *ε_j_* denote the sum of the position error for each estimated point *i* = {1*..k*}, in a given trajectory *j*, and let *ε_acc_* denote the average *ε* of the different sequences. At the same time, let $\bar{\varepsilon }$*_j_* compute the average position error for all the *k* steps in sequence *j*, and let $\bar{\varepsilon }$*_acc_* accumulate this same value on average for all the 10 sequences. The average error metrics in Table 1 show how the collaborative approach has a strong advantage over the classical approach in singular movements:

(15) $$\varepsilon_{j}=\sum_{i=1}^{k}\left(\left\|r_{i}^{WC}-{\hat{r}}_{i}^{WC}\right\|\right);\;\varepsilon_{acc}=\frac{1}{10}\sum_{j=1}^{10}\varepsilon_{j}$$

(16) $${\bar{\varepsilon }}_{j}=\frac{1}{k}\sum_{i=1}^{k}\left(\left|r_{i}^{WC}-{\hat{r}}_{i}^{WC}\right|\right);\;{\bar{\varepsilon }}_{acc}=\frac{1}{10}\sum_{j=1}^{10}{\bar{\varepsilon }}_{j}$$

All the error metrics observed produce noticeable lower values for the proposed approach, all them lower than half the classical DI-D approach metrics. As the drift accumulates, with locally long trajectories (without map splitting or similar approach), the error grows faster the longer it runs, so for both approaches we see that the final position error is notably over the average instantaneous error.

### 5.3. High Angular Speeds within Small View Spaces

**Figure 13:**
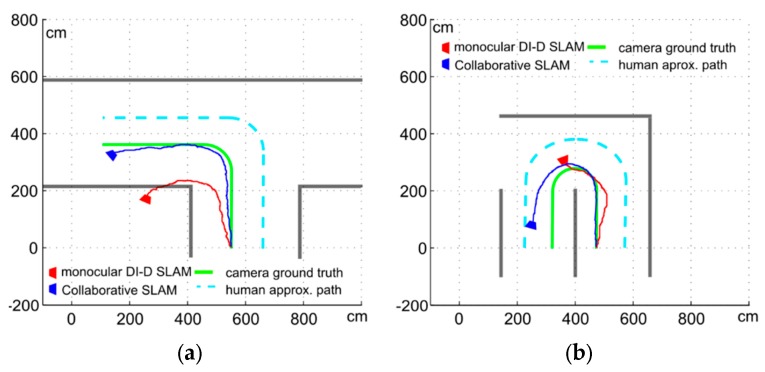
(**a**) Turning 90° and (**b**) 180° with monocular SLAM (red) and collaborative monocular SLAM (blue).

Another of the main issues observed in monocular SLAM approaches is that during turns the observable environment changes very quickly, with all the features available in the map are no longer seen in a matter of seconds. This problem is very present in the delayed feature initialization approaches: while the undelayed approaches will initialize landmarks with very inaccurate depth estimations, it is possible that a delayed approach is not able to find and initialize new features as quick as those in the map become no longer visible. When the number of features seen in an environment drops below a threshold (which depends on several factors, as the movement and rotation speeds, the quality of the detected features, *etc.*), the EKF loses convergence quickly, leading to completely distorted trajectories, or in some cases, estimated trajectories which do not match the actual ones even in direction. When combined with forward aligned movements w.r.t. the camera visual axis, turn and twist become an even worse issue (see Figure 13).

Figure 13 shows two experiments focused on turning. The robotic platform is traveling at 0.8 m/s and performs a 90° turn and a full 180° respectively, with the human following approximately the dashed blue line. In Figure 13a, the red trajectory shows how a pure monocular SLAM approach cannot really deal with a close turn, and the turn is overestimated. The trajectory estimation is further disrupted by the inability of the non-collaborative approach to fully deal with the forward camera depth movements. The final result overstates the turn by almost 80° and is not even able to keep the position estimation inside the corridors/observable environment.

In Figure 13b, the trajectories estimated for the 180° show with clarity the difficulty of turning for EKF based monocular SLAM procedures. The purely monocular approach simply ends losing convergence (thus not being able to process the complete sequence in a meaningful way) after losing the orientation estimation and turning sense. As before, the forward movements are shown to be especially unsuitable for monocular SLAM approaches. The collaborative approach (blue trajectory) is able to estimate most of the trajectory done in the sequence. It is worth noting that the position error, at 0.94 m, is almost as big as the case shown in Figure 13a, while the distance travelled is much shorter (about 6.65 m). Introducing the turn, even when the orientation can be considered as correctly estimated, with a final orientation of 21.4°, has increased the drift error, with a final position error proportionally more than twice bigger than in a straight trajectory.

### 5.4. General Trajectories and Performance

In order to further evaluate the gains and effectivity of the proposed technique, and specifically, the impact of the measurements with the pseudo-stereo procedure, a series of metrics have been developed. These metrics allow studying the effect of the periods where the overlap is available, taking into consideration factors such as the duration of the overlaps and their distribution. To test them and obtain relevant numbers, a more general sequence set, with both straight sections and turns has been captured.

The main interest is to study the interaction of the overlap periods with the gain in accuracy in the odometry estimation. With this objective, two different metrics are used to study the overlap periods distribution and duration, the *τ* overlap time regularity (Equation (17)) and the *κ* non-overlap time deviation (Equation (18)):

(17) $$\tau =N\sum_{i=1}^{N}\left(\left|\eta_{i}-\frac{\eta_{total}}{N}\right|\right),\text{ where }\eta_{total}=\sum_{i=1}^{N}\left(\eta_{i}\right)$$

(18) $$\kappa =\frac{1}{M}\sum_{j=1}^{M}\left({\left(\mu_{j}-\bar{\mu }\right)}^{2}\right),\text{ where }\bar{\mu }=\frac{1}{M}\sum_{i=1}^{M}\left(\mu_{i}\right)$$

The two coefficients represent the regularity of the separation between overlap periods (*κ*), and the In these expressions, N and M are the number of intervals with and without overlap respectively, with η_i_ being the duration of *i-*th interval with overlap, and μ_j_ the duration of the *j-*th interval without overlap. These expressions are only useful for cases with more than a single period, as they measure the relation between them, trying to identify whether certain overlap distributions provide more advantages. similarity between the duration of these overlapping periods (*τ*). In both metrics, the lower values, tending to zero, represent what is considered a better distribution of the overlap time (with the requisite that both M and N are greater than 1. A low *κ* value means that the intervals where overlap is present are distributed uniformly; while a lower *τ* value implies that these intervals of overlap are of similar duration, and that the overlap time is not concentrated mostly in a single period. An additional metric has been designed to evaluate the return rate of the computational overhead (*U*) supposed by actively following the proposed SLAM strategy. Equation (19) describes this value, which is based on the cumulative squared error of the position, but considering also the length of the trajectories and the duration of the overlap periods:

(19) $$U=\frac{1}{\left\|r_{k}^{WC}-r_{1}^{WC}\right\|}\cdot \sum_{i=1}^{k}\left({\left(r_{i}^{WC}-{\hat{r}}_{i}^{WC}\right)}^{2}\right)\cdot \eta_{total}$$

The duration of the overlap periods is introduced as a penalizing factor: if the squared errors are lowered by the use of the collaborative perception approach, they can offset the penalization, but if the improvements are low, *U* will grow. The inverse of the length of the trajectory is used as a normalizing factor: as the drift grows faster the longer the local trajectory is extended, the growth of the quadratic error and overlap penalization must be distributed along the whole trajectory.

**Figure 14:**
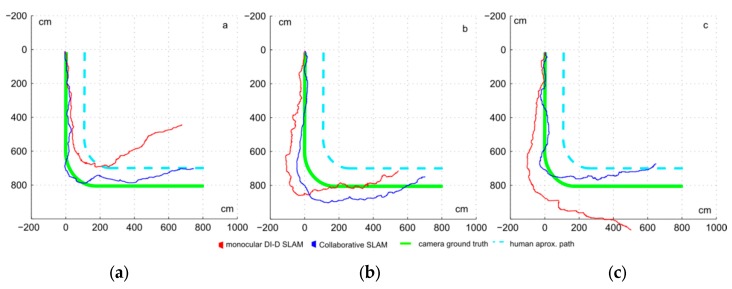
Trajectories estimated for classic DI-D and collaborative SLAM for Table 2. (**a**) case *a*, (**b**) case *b* and (**c**) case *c*.

**Figure 15:**
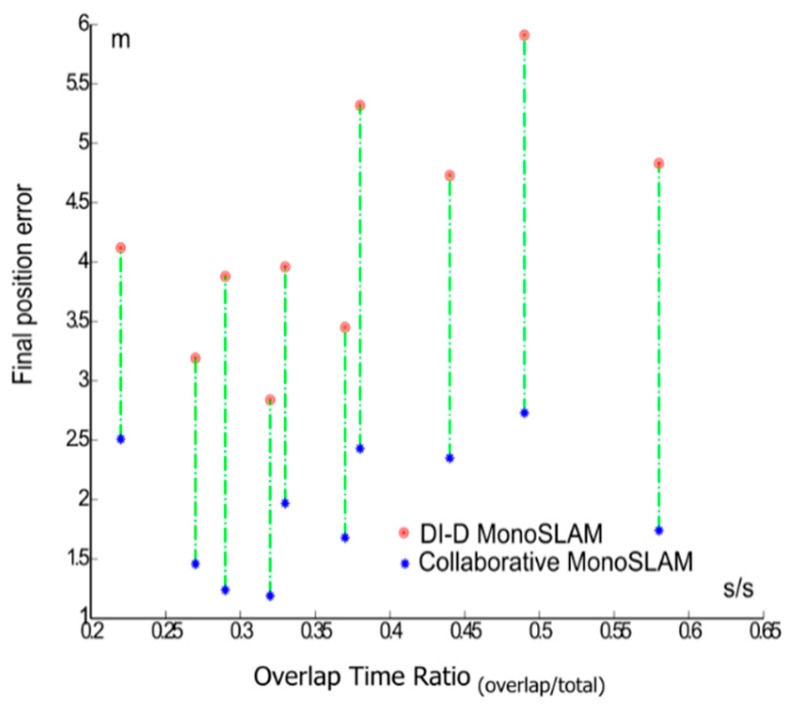
Final position error *versus* overlap time rate for sequences *a*–*j*.

The error and proposed metrics of the general set of sequences are shown in Table 2. Three examples of trajectories are shown in Figure 14. The introduction of the pseudo-stereo measurement to the initialization of features and update step produces a consistent improvement into the odometry estimation. On several cases at Table 2, like Figure 14c, it is observed how pure monocular SLAM cannot make locally long trajectories without further help, but the proposed approach helps improve the results notably. It is also worth noting that there may be correlation between the time where the pseudo-stereo measurement is available (noted as overlap time), and a decrease in the odometry error. Figure 15 plots the final position error for each sequence, with and without the introduction of pseudo-stereo measurements, linking the errors of each sequence, against the overlap time rate. In this figure it can be observed how the distance between the errors for the classic approach and the proposed approach grow as the overlap time ratio grows.

The proposed metrics, *κ*, *τ* and *U* produced mixed results. While *τ* showed no appreciable correlation between the regularity of the overlap periods and the different error metrics, *κ* exhibits some more relation between the results. While intuitively, splitting the overlap time in several periods in a spaced manner should be more convenient, as it reduces the covariance between the observed features and the camera to that uncertainty of the pseudo-stereo measurement, the data obtained is not conclusive enough to infer a correlation.

On the other hand, the *U* value offered insight and helped provide an analysis less focused on accuracy and centered on the costs of the pseudo-stereo measurements. The computational costs of the DI-D monocular SLAM have been already discussed in [31], and given a fixed maximum on the number of features, it can be assumed to be bound by an upper limit. Then it is logical to observe the other process with great computational costs associated, which is the introduction of pseudo-stereo measurements. The costs are incurred because the proposed technique requires to search for points of interest at one image and to compute SURF descriptors of two frames at each EKF iteration where is applied. Thus, the U value helps to keep in perspective the trade-off between accuracy and cost. On average, the additional overhead introduced by the pseudo-measurement procedure supposed less of a quarter of the total computational cost (about 23% of time) in the simple MATLAB implementation. Still, this cost could increase as the overhead is only incurred in 37% of the frames on average. In a worst case run, where the pseudo-stereo cost penalty is incurred for each frame (even when there is no overlap between fields of view), this penalty becomes almost the 45% of the time consumed. This increased computational cost could easily make the approach unmanageable in real time.

## 6. Conclusions

This paper describes a completed approach to the monocular SLAM problem, where data obtained from a human-deployed sensor is fully fused into the EKF SLAM methodology. The data produced by the secondary sensor allows converting the standard monocular measurements (detailing heading and attitude) into pseudo-stereo measurements, which also include the depth. These pseudo-stereo measurements are used in all the steps of the EKF, including the measurement and update step of the extended Kalman filter and the feature initialization, differing from previous works where only the feature initialization task [23] used the pseudo-stereo enhanced depth estimation. This implies that the pseudo-stereo measurement procedure has to be accounted for both on the direct and inverse observation models, as it can be used in both steps. While the C_f_ camera can move freely, a combination of data from the robotic sensors and the wearable devices allows estimating its pose with respect to the robotic camera C_s_. As further described in [23], although it is possible to perform a full stereo process based on epipolar geometry with the available data, the epipolar stereo estimation was rejected based on the image processing required for warping images according to the relevant homographies [10]. Thus, matching points with SIFT/SURF descriptors proved to be the most convenient approach.

One of the shortcomings in a previous work [23] was the utilization of a standard undelayed inverse observation model to compute the update of the covariance matrix once a new feature was introduced into the EKF. As the new approach required the formulation of new Jacobians to compute the Kalman gain and innovation covariance during the update step, some effort was put in updating the initialization process to use a more accurate representation of the process covariance, although its impact is thought to be small. The main contribution comes in the commented update step, where actual measurements with full depth obtained without delay have become available, instead of only measuring features in terms of pixel coordinates. This meant that the classical Kalman innovation formulation for pixel-based features needed to be updated. The new procedure builds the Jacobian ∇H once all the features have been correctly measured, in order to know if any given feature will be treated as pixels in camera frame coordinates or as real world 3-dimensional point. While delaying the construction of the Jacobian is slower than building it along the measurement process, as typically done in monocular approaches [6,7], it avoids the dynamic matrix resizing penalization incurred by having to refit a partially built Jacobian matrix.

An initial study in simulations allowed characterizing the gains and advantages of the approach with respect to the uncertainty in the feature measurements. The results of these simulations showed a high correlation between the uncertainty in the depth measurement and that of the state of the system, especially in terms of the camera position estimation.

The experimental sequences captured allowed to test the proposed methodology with real data. The main focus was evaluating the strengths of the proposed technique both as a general approach and specifically against the most troublesome scenarios for classical monocular SLAM, be it delayed or undelayed. Thus, several sets of sequences were captured: (i) those looking like a general indoor trajectory, and (ii) specific sequences with singular movements in mind, like those aligned with the depth axis of the camera, and close turns. For processing the sequences, no large map management technique was used, thus all the drift was accumulated over. These sequences show how the proposed approach has much more accuracy and resilience than ordinary monocular EKF SLAM. The forward advance sequences show clearly how monocular EKF SLAM has many troubles estimating the forward movement, thus producing a great underestimated odometry, with big orientation errors, while the proposed approach estimates the trajectory with greater accuracy, with an error in the range of 1 m for the best case. On the other side, the turning sequences showed that close turns are probably one of the hardest movements for monocular SLAM to estimate, to the point of completely losing convergence if quick enough. These claims are further proved by the computed error metrics.

For the experiments the collaborative SLAM approach was offline executed in a MATLAB implementation, thus time performance data would be unreliable, though previous works based on the same SLAM methodology performed robustly on real-time [7]. Additional computational overhead introduced by processing two images per frame when overlap is found and matching the SURF descriptors should be easy to deal with using parallel processing of the images within a strong implementation from a computer science point of view.

As the robustness and viability of the proposed approach has been probed experimentally, future works should deal with finding ways to obtain even more advantages from the HRI framework, and analysing and improving the system from a technical point of view. The impact of the depth measurement data could be studied as a function of its availability, overlap region size, and variance of the C_f_ pose estimation. Further study and development on the proposed metrics to evaluate the effectivity of the overlap in function of its distribution could enhance the method by predicting when an overlap measurement could be needed/most beneficial, and cueing this to the human component of the system. From a technical point of view, the MATLAB implementation can be studied so to produce an optimized version with real-time performance as the target. It is worth noting that as the trajectories have been completed within single local maps, the approach should prove to be robust enough to scale it to longer and more complex trajectories using submapping techniques related with map management and loop closing.

## Figures and Tables

**Table 1:** Average metrics for DI-D MonoSLAM and Collaborative Monocular SLAM.

Approach	Avg. Accumulated Position Error *ε_acc_* (m)	Avg. Instantaneous Position Error (m)	Avg. Final Position Error (m)	Avg. Overlap Time Ratio (s/s)
DI-D MonoSLAM	694	5.56	6.78	-
Collaborative MonoSLAM	276	2.21	3.17	0.387

**Table 2:** Metrics for Collaborative Monocular SLAM overlap time evaluation.

Sequence	DI-D SLAM Errors (m)		Collaborative MonoSLAM Errors (m)		Overlap Time Ratio (s/s)	*τ*	*κ*	*U*
	Final Position	Avg. Instant	Final Error	Avg. Instant Error				
*a*	3.88	3.17	1.24	0.82	0.23	1.9	1.4	33.8
*b*	2.84	2.00	1.19	0.69	0.32	1.1	0.7	36.9
*c*	4.12	3.04	2.51	1.72	0.22	1.4	1.2	126.2
*d*	3.19	2.26	1.46	0.98	0.27	2.2	2.9	54.3
*e*	5.32	4.12	2.43	1.73	0.38	3.4	2.5	223.1
*f*	3.45	2.15	1.68	1.28	0.37	2.8	3.3	115.6
*g*	4.83	3.34	1.74	1.12	0.58	4.1	1.8	159.1
*h*	3.96	2.87	1.97	1.34	0.33	1.7	2.3	122.4
*i*	5.91	4.43	2.73	2.26	0.49	1.6	0.8	316.7
*j*	4.73	3.92	2.35	1.54	0.44	3.8	3.2	232.5

## Appendix A: Nomenclature

- C_s_: SLAM camera
- C_f_: free camera
- T_c_: camera transformation matrix
- *x_i_, y_i_, z_i_*: camera coordinates
- *θ_i_, φ_i_*: camera azimuth and elevation
- *r_i_*: real depth to feature
- *ρ_i_*: inverse depth to feature
- *d_x_, f, ρ, (u0,v0)*: camera calibration parameters
- *(u_u_, v_u_)*: undistorted pixels
- σ_Cs_, σ_Cf_: angular measurement noise for C_s_ and C_f_
- σ_r_: depth measurement noise
- *d*: actual depth for a feature
- ε_j_: absolute error for sequence *j*
- ε_acc_: average ε_j_ for all the sequences
- $\bar{\varepsilon }$_j_: MAE for sequence j
- $\bar{\varepsilon }$_acc_: average $\bar{\varepsilon }$*_j_* for all the sequences
- η: duration of an overlap period
- μ: duration of a non-overlap period
- κ: non-overlap time deviation
- τ: overlap time regularity
- Ω: feature map
- **m**: unitary direction vector
- $\hat{x}$: augmented state vector
- ${\hat{x}}_{v}$: robot camera state vector
- **r***^WC^*: camera optical center position quaternion

## Appendix B

These are the equations describing the update step of the Extended Kalman Filter used (full details in [6]):

(B1) $${\hat{x}}_{k}={\hat{x}}_{k+1}+Wg$$

(B2) $$P_{k}=P_{k+1}-WSW^{T}$$

(B3) $$W=P_{k+1}\nabla H^{T}S^{-1}$$

(B4) $$S=\nabla HP_{k+1}\nabla H^{T}+R_{uv}$$

(B5) $$g=z-H\left(\hat{x}\right)$$

In this notation, the *k* index denotes the temporal condition of a given estimation or variables, thus, *k* denotes the last posterior estimation, or the last corrected data, and also those values based on this data; while *k + 1* denotes the last prior estimation, generally considered as the current prediction for any given iteration.

## Appendix C

**Table C1:** Sensor measurement specifications.

Sensor	Model and Manufacturer	Capture	Errors
Hean Worn:			
*Camera*	Logitech c170	Up to 1024 × 768, Up to 30 fps	Vertical: 1 pixel, Horizontal: 1 pixel
*AHRS*	Xsens Mti 30	Roll/Pitch/Yaw at 100 Hz	Roll/Pitch 0.5°, Yaw 1°
On Robotic Platform:			
*Camera*	Logitech c170	Up to 1024 × 768, Up to 30 fps	Vertical: 1 pixel, Horizontal: 1 pixel
*Frontal Laser RF*	Leuzer RS4-4	133 points, 190° arc, 25 Hz	1.43°, min. range 0.3 m, max. range 20 m
*Back Laser RF*	Leuzer RS4-4	133 points, 190° arc, 25 Hz	1.43°, min. range 0.3 m, max. range 20 m
